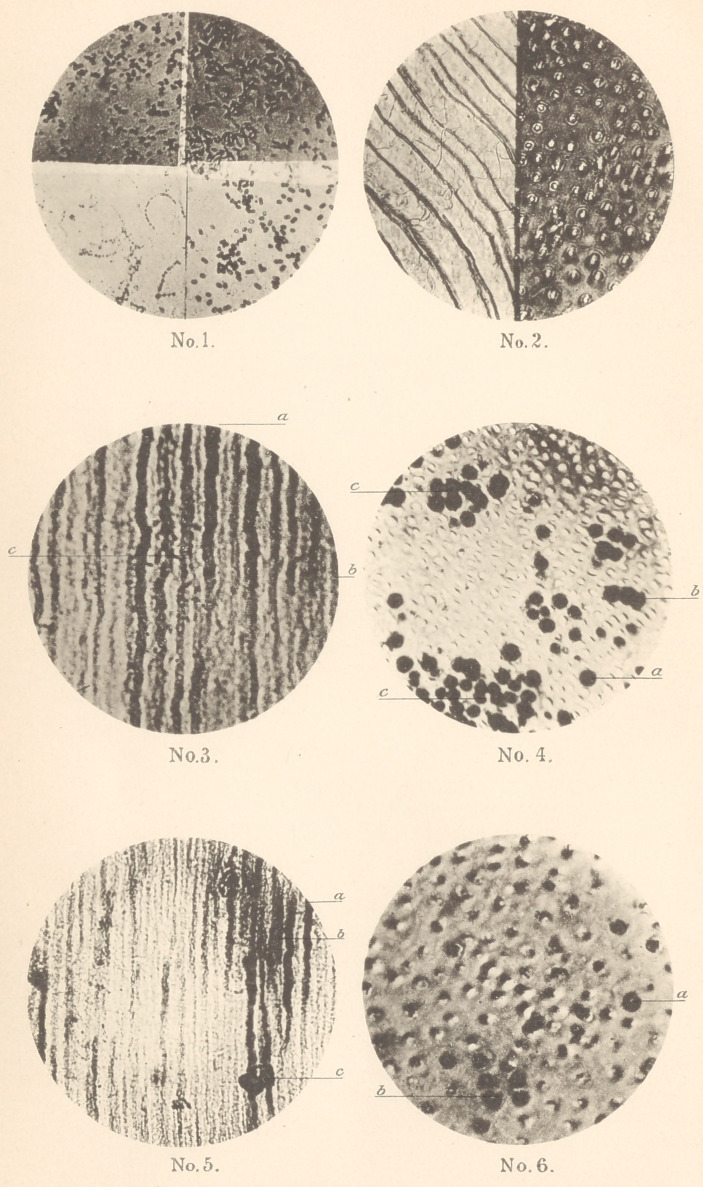# The Etiology of Dental Caries

**Published:** 1889-03

**Authors:** George S. Allen

**Affiliations:** New York City


### International Dental Journal.

VOL. X. MARCH, 1889. NO. 3.

### Original Communications.[1]

### THE ETIOLOGY OF DENTAL CARIES.[2]

BY GEORGE S. ALLAN, D.D.S., NEW YORK CITY.

*Mr. President and Gentlemen:*—It was with much reluctance I accepted the kind invitation to appear before you to-day with a paper on the subject announced. Lack of new material and time to redress old matter would seem to have been sufficient excuse for my hesitation, and then, again, you have probably had rather more of dental caries of late than you may think to be a proper allowance. I was, however, told not to write much and that a short summary would be all sufficient as an introduction to the lantern views which would interest greatly and would be new to most of you. With this understanding, I undertook the task.

Some able workers and writers think that in the main the problem of dental decay has been solved, and profess to be able to demonstrate their position—in fact, say they have demonstrated it, and point with much pride and certainty to the proofs. What are they? Let us as briefly as possible look at them.

In order to anticipate a certain class of objections let me here state that no reference will be made to the so-called-indirect-predisposing causes of dental caries, but I will admit them at once and give them a prominent place among the factors promoting tooth destruction. Crowded arches, imperfect and faulty development, cracks, fissures—*et id genus omne*—of promoting conditions,

[1] The editor and publishers are not responsible for the views of authors of papers published in this department, nor for any claim to novelty, or otherwise, that may be made by them. No papers will be received for this department that have appeared in any other journal published in this country. The journal is issued promptly on the 15th of the month.

[2] Read at the Tenth Anniversary Meeting of the Odontological Society of Pennsylvania, December 12, 1888.

do unquestionably invite decay, but they do not in any way produce it. Keep away the active agent or agents that destroy the teeth, and the most faulty set of teeth will last just as long as the most perfect. Alter the conditions by introducing the active agent or agents and at once all is changed. Where the opportunities for destruction are the greatest, there will decay proceed most rapidly, as a matter of course.

Practically all agree on two points: 1st. That dental caries invariably commences on the outside of the tooth; and 2d, that an acid or acids begins the work of tooth destruction. So much only is common ground. The radical differences lie (1.) in accounting for the presence of the acid in the oval cavity, and (2.) in estimating the resistive power of the tooth itself, whether it be dead or alive. The real bone of contention is whence comes the acid and why does it make cavities in tooth structure instead of uniformly eating it away on all surfaces.

I will call your attention to two theories only. The one has been denominated "The Germ Theory," the other, for want of a better term, I will call the "Protoplasmic Theory." Dr. Miller, of Berlin, is the author of the first, and Drs. Abbott and Heitzman, of New York City, of the latter. To the first I will especially invite our attention, for the reason that it is complete in all details, and thoroughly accounts for the conditions as found in the mouth. To the second I will refer only briefly to show you that it is fallacious from the foundation up.

I premise by saying that the germ theory is the only theory ever presented to the profession that is grounded on a demonstration from begining to end, and from which every possible source of error has been eliminated by the most careful and painstaking series of experiments. It accounts for nearly every phase of caries; shows whence comes the acid that first dissolves the lime salts of the teeth; clearly explains how the animal basis substance is afterwards destroyed and shows how, per force, cavities or pockets are formed in the teeth.

The actual presence of an acid as a commencement or initiative step in the process of decay being acknowledged Miller shows us whence it comes, names it and points out the little organism at work manufacturing it. All other investigations have depended on guess work mostly to account for it. In brief, it is lactic acid that does the work and it is one of the waste products of bacterial life in the presence of a fermentable substance. It is one of the so-called ptomaines. Only a few bacteria eliminate this acid in growing; but let it be produced and brought into contact with the lime salts of the tooth, chemical action at once takes place. The lactic acid supplanting the phosphoric and carbonic acids of the tooth and forming soluble salts. Fresh supplies of food for the bacteria are constantly obtained from the sugars or amylaceous matters in the mouth, and so each little crack or break in a tooth, or other spot difficult of access, and hard to keep clean, becomes a focus of destructive activity; a little acid manufactory as it were. Were it not for the constant absorption of the acid by the lime salts of the tooth forming lactate of lime, bacterial life, in a cavity, would soon cease. They would be smothered in their own waste products and die, as naturally as we would die were we compelled to remain in a close room in the presence of the waste products of our life, viz., the carbonic acid from the lungs, the urine from the kidneys and the fæces from the bowels; but the lime salts act the part of scavingers for the bacteria and keeps their garden in good condition. The acid first formed commences the cavity, and as solution of the lime salts takes place the bacteria follow after, penetrating and enlarging the dentinal tubuli.

In advance of the bacteria, there is always to be found a zone of semi-decalcified dentine. Exactly what the ptomaines is that completes the work of tooth destruction, breaks down the animal basis substance, is yet to be determined; but it is also a waste product of bacterial life. Up to the date of the publication of Dr. Miller's researches and experiments, the constant presence of micro-organisms in the carious dentine was generally recognized and some writers had even more than suspected that they played an important part in tooth destruction; but is was the good fortune of Dr. Miller to be able to demonstrate the correctness of the suspicion and to remove all doubts by showing how they did their work and from whence they derived their power.

So far, the labors of Dr. Miller might be called analytic; but he went further and synthetically silenced opposition. He took a freshly extracted, healthy, bi-cuspid tooth and sterilized it completely by heat, employing about 300° or 320° Far. All germs, or spores of germs, were thus destroyed. The tooth thus treated was cut into small pieces—some very thin—placed in a tube containing a pure cultus of the bacteria, constantly found in decaying teeth, in the presence of a non-fermentible fluid, and some in the same fluid to which a fermentible substance had been added—beef extract being the former, and beef extract plus two per cent. of cane sugar, the latter. .Both tubes were then placed in a suitable warm chamber and the effects noticed. In the first, though the bacteria flourished, no change was observed in the portions of tooth placed therein. There being no fermentation, no acid was found; in the latter, however, a pronounced action was apparent in a few days, and the pieces on being removed from the fluid were found to be soft and pliable and could readily be cut with a knife or razor. Two or three weeks maceration showed that disintegra. tion and destruction of the substance of the tooth was rapidly going on. Thus far in all respects, so far at least as the first stages were concerned, the action was similar to that observed in using a weak solution of chromic acid—the acid commonly employed in the laboratory for softening teeth, preparatory to section cutting. The sound healthy pieces of teeth thus decalcified were transferred to an ordinary freezing microtome and sliced, stained in the usual way and mounted in balsam, and then placed under the microscope for examination. The preparations thus obtained were found to resemble in a most marked manner those made from natural caries. In fact, so close was the resemblance, that the most expert microscopist could not tell the one from the other. In all essentials they were counterparts. In both are to be found the germs in the distended tubules. In each there is the same breaking down of the matrix and the formation of pockets or caverns by the fusing together of the tubules and bacilli. In each there is to be found, in advance of the germs, the zone of semi-decalified dentine. To this last fact I would draw special attention, it being of vital importance in comparing the two. Thus, a complete demonstration of the bacterial origin of caries was established. A preparation in my possession might be called the caries puzzle. It contains three slices of carious dentine. One, natural, from a living tooth; one, ditto, from a dead tooth, and the third, artificial caries. All have the same characteristics and it is impossible to tell them apart.

In artificially produced caries there is no possible explanation of the result brought about other than that it is due to lactic acid generated by the growth of the bacteria in the fermentable mixture into which the portions of sound teeth had been placed. On the screen, this evening, I will have the pleasure of showing photomicrographs taken from both natural and artificially produced caries, and you will be able to judge for yourselves how accurate my statements are. You will, at the same time, see some specimens of the finest expert work in lantern slides, made with high power objectives, in the world; and will with me give all praise and commendation to our good friend and our honored brother, Dr. Andrews, of Cambridge, for his most successful efforts in our behalf. Lately, I have seen a letter from Dr. Miller, who pronounces them unequalled.

So much for the first division of my subject. As to the second, Dr. Abbott published in the *Cosmos*, in 1879, a series of papers on caries of the human teeth, to which now, though it may seem rather tardy, I wish to take exception, not, however, for the first time. I would not at this late day call your attention to it, but for the fact that more than one effort has been made of late to keep it alive, especially at the union meeting at Louisville last August; a full report of which was published in the *Cosmos* and INDEPENDENT PRACTITIONER. I must ask the author's pardon for giving a new name to his theory, still I think the one I have chosen, "protoplasmic theory," is most appropriate; and I believe all will agree with me, and for this reason: Dr. Abbott himself says, vide *Cosmos*, Vol. XXI, pages 57, 58:

"Before entering upon the consideration of the subject, however, I wish briefly to recapitulate what has recently been discovered by Dr. C. F. W. Bödecker in the minute structure of human teeth. The reasons why I do so are, that not only my own researches are corroborative of Bödecker's discoveries, but a full understanding of the morbid processes is possible only upon a correct knowledge of the normal conditions.

"The dentine is traversed by innumerable canaliculi, which ramificate both toward the enamel and the cement. Each canaliculus contains a delicate fiber of living matter, which is in direct connection with the protoplasmic formations within the pup cavity, with offshoots of the cement corpuscles, and the fibers between the enamel rods. Every dentine fiber sends innumerable delicate conical threads through the cavity of the canaliculus into the basis substance between the canaliculi, where a very minute network of living matter is present, uniting the dentinal fibers with each other throughout the whole tissue of the dentine. The basis substance is analogous to that of bone, therefore glue-giving, and at the same time infiltrated with lime salts. Around each dentinal canaliculus the basis substance is denser than between the canaliculi."

As that portion of the text referring to the enamel is rather long, and as the theory from its frequent repetition is well understood by the profession, a brief statement of its nature only will be given. It is that throughout the enamel, between the rods and running across them, there is to be found a reticulum of living protoplasmic threads and that this reticulum is in direct connection with a like formation in the basis substance of the dentine and cementum, and through these latter with pulp and circulatory system.

The meaning of the above is that the protoplasmic net-work theory of Drs. Bodecker and Heitzman is the basis on which Dr. Abbott builds his theory of dental caries. A word or so about this protoplasmic theory, to begin with will be appropriate.

Now I take it that a theory as far-reaching as this is should have a deeper and broader foundation than the *ipse dixit* of one, two or even three men. It should be capable of substantial proofs, and the proofs should be forthcoming to any intelligent, careful observer asking for them. The more so if they give a new dress to an old friend, and change his shape so that we fail to recognize him. Let me state, unhesitatingly, that the beautiful pictures exhibited by Dr. H. (vide *Cosmos*, Vol. XXIX, page 259) cannot be exhibited under the compound microscope, and are without existence, so far as their slides are concerned, in either enamel or dentine; and I here challenge them to produce any preparations in proof of their theory that I cannot, by the first microscopists in the country, show to be without value for their support.

So much for the protoplasmic theory, now as to Dr. Abbott's theory of caries which may be found in the *Cosmos*, Vol. XXI, page 58, and which is briefly summed up as follows: caries is an inflammatory process which begins by a chemical disintegration of the tooth substance, the irritating action of which produces a reaction upon the living matter, protoplasm, in the tooth. The micro-örganisms, which the author freely admits, are a constant accompaniment, are the sequence, and not the cause of caries. The main cause, according to Dr. Abbott, is a vital action, and the disintegration of tooth substance is due to a retrograde metamorphosis in the tissues.

It would be easy to multiply arguments as to its improbability from the nature of protoplasm itself, but I content myself here with the simple assertion that the preparations do not show what he says they show, and fail to substantiate in any degree his theory.

Allow me once more to refer to the specimens themselves. Shortly after listening to Dr. Abbott's paper, I applied to him for a loan of his preparations, as I wished very much to see and study them. Eight or ten were most kindly placed at my disposal, and I took them home to my microscope. This was some ten years ago. My disappointment was great. Let me here state that I went to work on them with a mind unbiased and ready to accept any truth they might show me. What I saw was simply this: overstained specimens; broken-down tooth-substance in which it was simply impossible to differentiate the elements or tissues; masses of micro-organisms and debris of all kinds, but none sharply defined. Is this an overdrawn picture? If it is, let him produce his slides to disprove it.

I want to go into the subject a little further and from another standpoint.

Let me draw your attention to the paragraph in Dr. Abbott's article in which he attempts to account for the presence of the acids that first attack the teeth. The attempt is a surmise or suggestion only, based on no experiments or proofs whatsoever. It is that, in the main, they are generated from the decaying material retained in exposed places in the teeth, and the most important role is given to meats in their various stages of putrefaction. Now, as a fact, free lactic acid can only be derived from meats by the conversion of sarcolactic, which is more or less a constant element in muscular tissue, into lactic, by the aid of some ferment. But the amount of acid which can be accounted for in this way is exceedingly limited, and by no means meets the wants of the case, and we cannot but think it would have been wiser on the part of the writer had he omitted it. Mills and Underwood, in their experiments, subjected sound teeth to the action of the products of putrefaction in meat for weeks and months, but without perceptible results, and finally abandoned their work in disgust, and gladly threw away their material. Another point, appealing at once to the daily observation of you all against the idea that putrefactive conditions can affect the teeth, may be drawn from dead teeth in which the pulp has been allowed to remain a length of time, exposed to the fluids of the mouth. Here you have putrefaction pure and simple; but you do not find it accompanied by caries, although everything is favorable: dead, rotting animal tissues in immediate contact with tooth-substance. It should be found here, if anywhere: that is, if putrefying animal matter ever produces caries. There can be then not the slightest foundation for this supposition—not enough to warrant attention; and, even if it were so, alkalies are also formed at the same time, and in quantity more than sufficient to neutralize any free acid.

It would be impossible, without either preparations or lantern slides made from them, to go into anything like a careful analysis of Dr. Abbott's paper; so I hasten on to the general summary. He says:

"In enamel, caries in its earliest stages is a chemical process. After the lime salts are dissolved out and the basis substance liquefied, the protoplasm reappears and breaks apart into small, irregularly-shaped, so-called medullary or embryonal bodies." In sober earnest, let me ask you how can this be? According to Dr. Heitzman, in living enamel the protoplasmic threads are exceedingly minute, and chemical analysis proves that the actual amount of animal matter in it is only some four per cent. at the maximum. How then can the protoplasm reappear? Either the great bulk of the enamel is but an allotropic condition of protoplasm—and therefore enamel is protaplasm pure and simple, and needs but the vivifying presence of an acid to manifest itself, or else the small amount contained in the threads must grow prodigiously. But whence can it draw its nourishment? Not from the blood, for the tooth, dentine and enamel is non-vascular, and the Doctor certainly would not say the nourishment came from the decomposed food and matter contained in the cavity? If he suggested such a thought, he would at once destroy his own theory. But his expression is, "the protoplasm reappears." Then it never could have been dead, or otherwise it would not be the original protoplasm. Here we face another difficulty. In all the various papers published by Drs. A. B. and H., they take the ground that both dentine and enamel are formed by the direct conversion of the odontoblasts on the one side into dentine, and of the ameloblasts on the other into enamel. It would seem then—I want to be careful in my expressions, that these tooth-forming cells, having been converted or transformed into something else—something certainly different in its nature from the original protoplasm, would find some difficulty in returning to their original medullary or embryonal condition. The puzzle is too great for me. So much for their theory of decay as found in enamel: Now what of that found in dentine? of which Dr. A. says:

"Caries of dentine consists in a decalcification, and in turn a dissolution of the glue-giving basis-substance around the canaliculi as well as between them. The living matter contained in the canaliculi is transformed into nucleated protoplasmic bodies, which, together with protoplasmic bodies originating from the living matter in the basis-substance, form the so-called indifferent or inflammatory tissue." (See *Cosmos*, vol. xxi, p. 179.)

"Caries of a living tooth therefore is an inflammatory process, which, beginning as a chemical process, in turn reduces the tissues of the tooth into embryonic or medullary elements—evidently the same as, during the development of the tooth, have shared in its formation—and in its development and intensity are in direct proportion to the amount of living matter they contain, as compared with other tissues." (See *Cosmos*, vol. xxi, p. 179.)

A similar condition to that found in enamel, but more pronounced, is thus stated by Dr. Abbott to exist in caries of dentine. As there is no question as to the relatively larger amount of animal matter to be found in the dentine compared with enamel, we would naturally expect to find in the dentine more positive proofs of inflammatory conditions provided any such existed; but, as I stated before, my microscopic examination of his slides utterly failed to substantiate his theory. No such thing as medullary or embryonal elements could be distinguished.

I would draw your attention to the fact that both enamel and dentine are non-vascular and not influenced by the circulation; therefore no true inflammatory conditions can exist. Search your works on pathology from beginning to end, and you will fail to find any inflammations described that are not in a greater or less degree dependent on the circulation for their inception and continuance. Cut off any organ, or a part of the body, from the circulation and you cannot, in such organ or part of the body, bring on inflammatory changes. Decomposition and death will follow, but unaccompanied by inflammatory conditions. Dr. Abbot admits this when he says, under the title of aphorisms: *Cosmos*, vol. xxi, page 180.

"The medullary elements, *owing to want of nutrition* and to continuous irritation, become necrosed, and the seat of a lively new growth of organisms common to all decomposing organic material." The italics are mine.

Of course, so far as the transmutation of the bases substance of the dentine into embryonal or medullary elements is concerned, the same difficulties exist as in the case of enamel; nor are they a whit less powerful. Embryonal elements can only be derived from pre-existing embryonal elements, and such growth can only take place in the presence of the proper conditions of food supply and life activity. Both of which are here absent. It is true the contents of the tubuli is living matter, but hardly embryonal or protoplasmic. It is matter that has been derived, formed, from protoplasm. The effect of the acids producing decay would be to destroy living protoplasm as a first effect, and the plugging up of the tubules by micro-organisms, acknowledged by Dr. Abbott to be universally present, would cut off all possible connection between the living pulp and the seat of decay. Think of it a minute what are we called upon to accept as sound pathology. A dense, mineral tissue formed, as they say, by the direct conversion of embryonal tissue is acted on by an acid, which dissolves the mineral elements and immediately it is changed into its antecedent protoplasm. Dead matter changed into living matter, and that without the intervention of living matter. The whole theory is certainly new and depends on new laws to sustain it. In the whole domain of pathology we have nothing like unto it.

At first sight it would seem strange that the phenomena attending caries of dead pulpless teeth should receive so little consideration at the hands of the founders of the inflammatory theory. Dr. Abbott alludes to them only incidentally, making them of secondary importance. In one place he says : " The decay of artificial teeth, either human or ivory, in all probability runs either an acute or chronic course, according to the amount of lime salts infiltrated into the glue-giving basis substance." In aphorism No. 8, he says : " In dead and artificial teeth, caries is a chemical process assisted only by the decomposition of the glue-giving basis substance of dentine and cement." In the main, he would have us look upon caries in these cases as of a chronic nature. The truth is that there are no marked differences discoverable in the carious process whether it attacks either living or dead teeth. In both cases, there is the same formation of cavaties, relatively the same rates of progress, and under the microscope, in prepared specimens, the same display of distended tubules filled with micro-organism; the same advanced zone of semi-decalcified tooth substance preceeding the germs, and the same cavernous breaking down of tooth substance as the animal glue-giving basis substance melts down under the continued action of the bacteria-produced ptomaines. These facts alone would seem to furnish an all-sufficient answer as to whether the inflammatory theory ought to live or die.

Summing up I would say that the germ theory is the only one so far that clearly and satisfactorily accounts for the acid; the prime agent in tooth destruction. The inflammatory theory simply begs this question or offers a lame hypothesis.

The germ theory alone can repeat out of the mouth the processes that go on within the mouth, and produce an artificial caries simulating perfectly the natural caries of the teeth.

The germ theory fully explains the distended tubules always found in dental caries and the broken down basis substance. The inflammatory theory is silent as to the distended tubules, and might as well be as to the breaking down of the basis substance and the formation of cavities in the dentine of diseased teeth.

### DESCRIPTION OF PLATE FOR DR. ALLAN'S ARTICLE ON DENTAL CARIES.

The photographs from which this plate was made were taken by Dr. R. R. Andrews, of Cambridge, Mass., and are wonderfully clear and distinct considering the power employed. They have not been equaled so far as the writer knows. The process employed for duplicating them (photo-gravure) simply reproduces the originals, and gives exact representations of the tissues without retouching, or any change whatever. All personal equations are to a certainty, therefore, removed. Many points had to be thought of in selecting the photographs; the purpose being to combine in one plate, as far as possible, a pictorial history of dental caries. To accomplish this some things most interesting had to be left out, and a method of arrangement adopted not as satisfactory as would have been possible had more cuts been allowed. This will be particularly noticeable in the groupings of the bacteria in the first figure, four plates being combined in one, and in the apparent lack of physical similarity between the figures of natural and artificial caries in long section. It would be easy to duplicate Figure 3 from artificial caries slides. It would also be quite easy to find the counterpart of Figure 5 from natural caries; but it seemed wiser to show to the two varieties rather than to seek for exact similarities. Figures 4 and 6, however, show the same characteristics. The extra shading in No. 6, between the distended tubules, is owing entirely to the mode of preparation of the slide; 4 was taken from a single, stained slide; 6 from a double stained one; 3 and 5 are practically alike, the bulbous expansions (*c*) in 5 alone differing from 3; but, as stated, these same bulbous expansions are found in natural caries, and are due to peculiarities in the teeth affected, not to any differences in the process. To fully appreciate the plate the reader should refer to some of Dr. Miller's original articles.

No. 1, x 1200. As it was found difficult, except in one or two solitary instances, to photograph the bacteria in situ, pictures were taken from the pure cultures of the bacteria, and four of these are here grouped together.

No. 2, x 1200. Cross and longitudinal sections of healthy dentine, taken where the tubules attained their maximum diameter. These pictures are extra fine, and show beautifully (1) the diameter of the tubules; (2) the thickness of the sheath of Neuman; and (3) the relative amount of space occupied by tubules and their contents to the inter-tubular dentine.

No. 3, x 1200. A long. section of dentine in a case of natural caries taken from the periphery of the teeth: (*a*) single, distended tubule; (*b*) two or more fused together; (*c*) one of the branching canaliculi, also infiltrated with bacteria.

No. 4, x 1200. Cross sections; natural caries: (*a*) three tubules fused together; (*b*) single, distended tubule; (*c*) tubules fusing together to form a cavern or pocket.

No. 5, x 600. Long. sections; artificial caries. It will be noticed that this picture has only half the amplification of the others: (*a*) single tubule enlarged; (*b*) two fusing together; (*c*) a bulbous expansion in two tubules. Whether these expansions are caused by the walls of the tubules at these points being weaker than at others, or by a plugging up of the tubules in some way, it would be impossible to say.

No. 6, x 1200. Transverse section; artificial caries: (*a*) single enlarged tubule; (*b*) four tubules fusing together. The shading between the tubules is a photographic effect due to the double staining.

## Figures and Tables

**Figure f1:**